# Melatonin potentiates the cytotoxic effect of Neratinib in HER2^+^ breast cancer through promoting endocytosis and lysosomal degradation of HER2

**DOI:** 10.1038/s41388-021-02015-w

**Published:** 2021-09-23

**Authors:** Zundong Liu, Xiaolin Sang, Min Wang, Yichao Liu, Jiao Liu, Xuefei Wang, Pixu Liu, Hailing Cheng

**Affiliations:** 1 Cancer Institute, The Second Hospital of Dalian Medical University, Dalian, Liaoning China; 2 The Second Hospital of Dalian Medical University, Institute of Cancer Stem Cell, Dalian Medical University, Dalian, Liaoning China

**Keywords:** Breast cancer, Targeted therapies, Cell signalling, Endocytosis

## Abstract

Complete blockade of the HER2 protein itself and HER signaling network is critical to achieving effective HER2-targeted therapies. Despite the success of HER2-targeted therapies, the diseases will relapse in a significant fraction of patients with HER2^+^ breast cancers. How to improve the therapeutic efficacy of existing HER2-targeted agents remains an unmet clinical need. Here, we uncover a role of Melatonin in diminishing HER2-mediated signaling by destruction of HER2 protein. Mechanistically, Melatonin treatment attenuated the protective effect of the HSP90 chaperone complex on its client protein HER2, triggering ubiquitylation and subsequent endocytic lysosomal degradation of HER2. The inhibitory effect of Melatonin on HER2 signaling substantially enhanced the cytotoxic effects of the pan-HER inhibitor Neratinib in HER2^+^ breast cancer cells. Lastly, we demonstrate that dual inhibition of HER2 by combined use of Melatonin and Neratinib effectively blocked the growth of HER2^+^ breast tumor xenografts in vivo. Our findings shed light on the potential use of Melatonin in a novel dual HER2 blockade strategy for HER2^+^ breast cancer treatment.

## Introduction

Amplification/overexpression of human epidermal growth factor HER2/ERBB2 is present in 20% of breast cancer. To date, HER2 positivity remains the only biomarker with demonstrated clinical utility in anti-HER2 targeted therapies [1]. Several classes of HER2-targeted agents have been developed for the treatment of HER2-positive (HER2^+^) breast cancer, including monoclonal antibodies (Trastuzumab and Pertuzumab), small molecule tyrosine kinase inhibitors (Lapatinib and Neratinib) and antibody-drug conjugates (trastuzumab emtansine T-DM1) [1–4]. While the clinical application of HER2-targeted agents has greatly improved the prognosis of HER2^+^ breast cancer, the diseases will inevitably relapse. The effectiveness of HER2-targeted treatments relies on more complete blockade of the HER2 protein itself and HER signaling network [1, 4]. Combinatorial HER2 blockade by combining different classes of HER2-targeted agents has demonstrated superior anti-tumor activity to anti-HER2 monotherapy in the preclinical and clinical settings [2–4]. However, how to improve the efficacy of existing therapeutics and identify more effective combinatorial strategies remains a pressing need for HER2^+^ breast cancer.

Neratinib, a next-generation irreversible small molecule inhibitor of receptor tyrosine kinases (HER1/EGFR, HER2, and HER4), has been recently approved for the treatment of HER2^+^ breast cancer [3–8]. Neratinib is a more potent inhibitor than the dual EGFR/HER2 tyrosine kinase inhibitor (TKI) Lapatinib, potentiating the cytotoxic effect of trastuzumab in HER2^+^ breast cancer cell lines [3, 9]. It has been reported that Neratinib has the ability to overcome Lapatinib resistance caused by incomplete inhibition of HER kinases [9, 10]. However, several mechanisms of resistance to Neratinib have been reported including dysregulated BCL2 family member expression and increased activity of Neratinib metabolizing enzyme cytochrome P450 CYP3A4 [11, 12]. In addition, somatic activating HER2 mutations have been detected in HER2^+^ breast cancer patients subjected to ongoing SUMMIT “basket” trial of Neratinib (NCT01953926), suggesting hyperactivation of HER family kinases may confer resistance to Neratinib [13]. There is thus an urgent need to identify novel targeted therapeutic combinations to improve the efficacy of Neratinib by abrogating the aberrant expression or activation of HER2 in the treatment of HER2^+^ or HER2-mutated breast cancer.

Melatonin is a natural hormone secreted by the pineal gland of human and mammals. Numerous studies including our recent work have reported Melatonin exhibits antitumor properties through diverse mechanisms of action [14–20]. In the current study, we investigated the potential effect of Melatonin as single agent and in combination with Neratinib in the treatment of HER2^+^ breast cancer cell lines. Interestingly, our study reveals an unexpected role of Melatonin in dysregulating HER2 protein stability and potentiating the cytotoxic effect of Neratinib in HER2^+^ breast cancer.

## Results

### Melatonin treatment recapitulates the effects of HER2-targeted agents

**Fig. 1:**
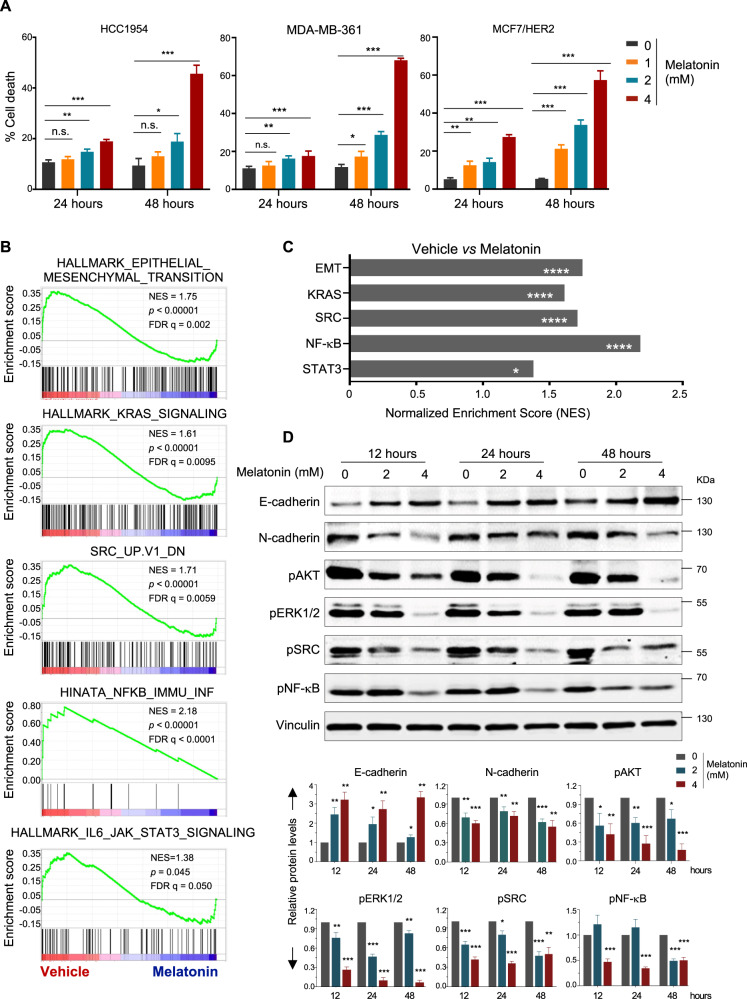
Melatonin inhibits HER2 downstream signaling in HER2+ breast cancer cells. **A** Cell death in the HER2^+^ breast cancer cells treated with Melatonin was determined by PI staining and FACS analysis. Drug treatment conditions were shown as indicated. Quantitation for three independent experiments is shown. Data are shown as Mean ± S.D. **B** Gene Set Enrichment Analysis (GSEA) of gene sets associated with Melatonin treatment in HCC1954 cells. Melatonin, 2 mM, 24 h. Normalized Enrichment Score (NES), *p* value and FDR q values of the correlation are shown. **C** The quantification of signatures downstream of HER2 as shown in **B**. **D** Western blot analysis of HER2 downstream signaling proteins in HCC1954 cells treated with Melatonin. Drug treatment conditions were shown as indicated. Vinculin was used as a loading control. The quantification of protein abundance is shown. Data are representative of three independent experiments. n. s., not significant. *p < 0.05, **p < 0.01, ***p < 0.001 (Student’s *t* test).

We first examined the effect of Melatonin on cell survival in three HER2^+^ breast cancer cell lines that carry oncogenic PIK3CA mutations, including HCC1954 (HER2 amplification, PIK3CA H1047R) and MDA-MB-361 (HER2 amplification, PIK3CA E545K), and MCF7 (PIK3CA E545K) with ectopic overexpression of HER2 (MCF7/HER2). Melatonin treatment resulted in significantly increased cell death in a time- and dose-dependent manner (Fig. 1A and Supplementary Fig. 1A). To understand the cytotoxic effect of Melatonin on HER2^+^ breast cancer cells, we recently performed transcriptome profiling of the HCC1954 cells treated with or without Melatonin by RNA sequencing (RNA-Seq, GSE175906). The gene set enrichment analysis (GSEA) revealed that Melatonin treatment was significantly inversely associated with enrichment of gene sets including EMT, KRAS, SRC, NF-κB, and IL6-JAK-STAT3 (Fig. 1B, C, Supplementary Fig. 1B), all of which have been previously linked to the therapeutic effects of HER2-targeted therapies [2, 21–24]. Consistent with these findings, Melatonin treatment led to markedly decreased phospho-AKT, phospho-ERK, phospho-SRC signals, and phospho-NF-κB signals, as well as attenuated EMT signature (reduced N-cadherin and increased E-cadherin expression) in HCC1954 cells (Fig. 1D). The observation that Melatonin treatment recapitulates many effects caused by HER2-targeted agents prompted us to investigate whether Melatonin may influence the expression and/or activity of HER2 itself.

### Melatonin decreases HER2 protein stability

**Fig. 2:**
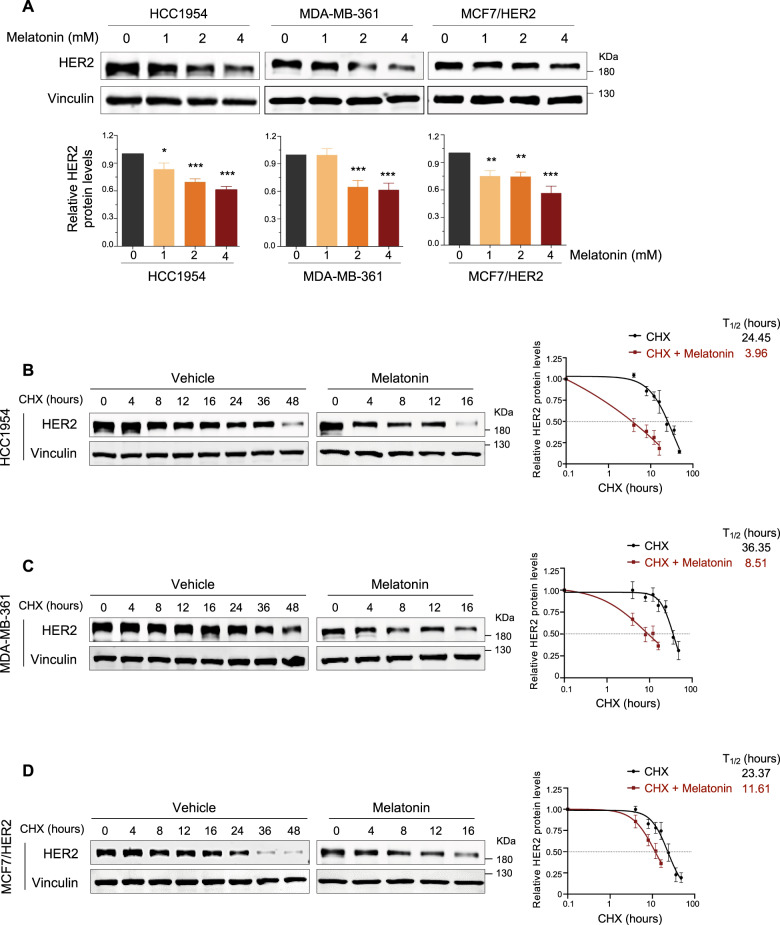
Melatonin decreases the stability of HER2 protein in HER2+ breast cancer cells. **A** Western blot analysis of HER2 protein levels in the HER2^+^ breast cancer cells treated with Melatonin at the indicated concentrations for 24 h. Western blot analysis of HER2 proteins levels in the cells treated with Vehicle control or Melatonin for 24 h followed by the addition of cycloheximide (CHX) treatment for the indicated time. Melatonin, 2 mM. CHX, HCC1954 (**B**), 20 μg/ml; MDA-MB-361(**C**), 40 μg/ml; MCF7/HER2 (**D**), 150 μg/ml. Vinculin was used as a loading control. The quantification of HER2 protein abundance and half-lives are shown. *T*_1/2_, half-lives. Data are representative of three independent experiments. *p < 0.05, **p < 0.01, ***p < 0.001 (Student’s *t* test).

We next investigated whether Melatonin regulates HER2 protein levels. Indeed, HER2 protein abundance is markedly reduced in all three cell lines treated with Melatonin (Fig. 2A). To examine whether Melatonin decreases HER2 through downregulation of its transcription, we conducted quantitative reverse transcription PCR (qRT-PCR) analysis. Melatonin treatment did not downregulate HER2 mRNA levels (Supplementary Fig. 2), suggesting that Melatonin-induced reduction at HER2 protein levels cannot be explained at the mRNA level of HER2.

Next, we asked whether Melatonin may affect HER2 protein stability. For this, we examined the effect of Melatonin on existing HER2 protein pools in HER2^+^ breast cancer cells using the protein synthesis inhibitor cycloheximide (CHX). As expected, the HER2 protein level gradually decreased in the vehicle-treated cells after CHX treatment in a time-dependent manner. In contrast, more striking degradation of HER2 was observed in the Melatonin-treated cells (Fig. 2B–D). Together, these results suggest that Melatonin treatment decreases HER2 protein stability.

### Melatonin treatment destructs HER2 through promoting its endocytosis and lysosomal degradation

**Fig. 3:**
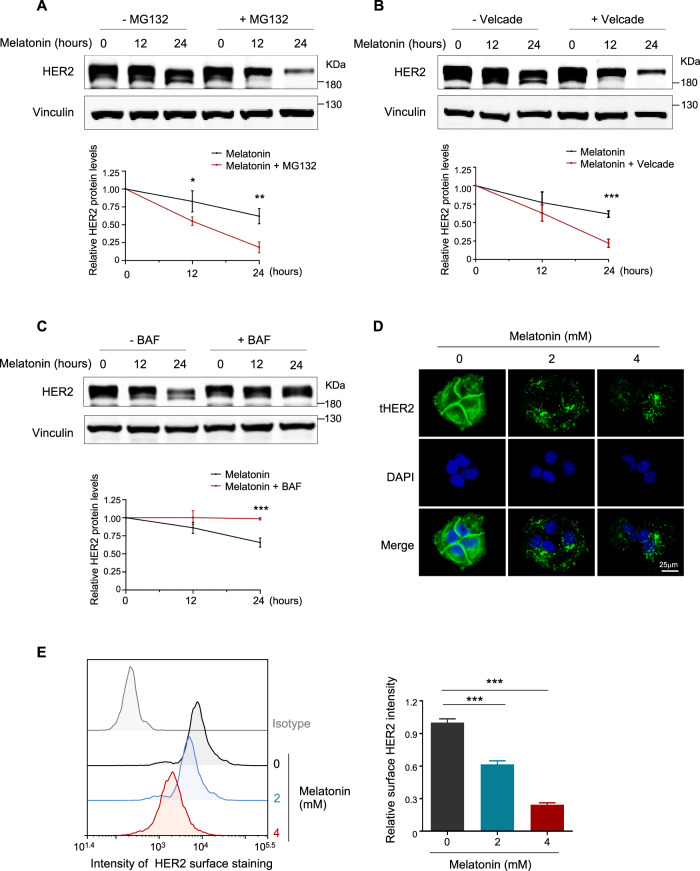
Melatonin decreases HER2 protein levels through lysosomal degradation. HCC1954 cells were treated with proteasome inhibitor MG132 (**A**) or Velcade (**B**) for 0.5 h or DMSO as control followed by the addition of Melatonin (0, 12, and 24 h). Western blot analysis and quantification of HER2 protein levels were shown. Melatonin, 2 mM; MG-132, 10 μM; Velcade, 500 nM. Vinculin was used as a loading control. **C** HCC1954 cells were treated with lysosome inhibitor BAF for 0.5 h or DMSO as control followed by the addition of Melatonin (0, 12, and 24 h). Western blot analysis and quantification of HER2 protein levels were shown. Melatonin, 2 mM; Bafilomycin A1 (BAF), 20 nM. **D** Representative images of immunofluorescent staining of HER2 in the HCC1954 cells treated with Melatonin at indicated concentrations for 24 h. Scale bar, 25 μm. **E** Flow cytometric analysis of HER2 protein levels on the surface of cells as in **D**. Quantification of HER2 abundance is shown as Mean ± S.D. Data are representative of three independent experiments. *p < 0.05, **p < 0.01, ***p < 0.001 (Student’s *t* test).

Receptor stability is known to be regulated by ubiquitin-proteasomal and/or lysosomal mechanisms [25–28]. We next sought to understand how Melatonin mediates HER2 downregulation. Interestingly, we found that proteasome inhibition by either MG132 or Velcade further reduced HER2 protein abundance upon treatment with Melatonin (Fig. 3A, B). In contrast, the lysosome inhibitor Bafilomycin A1 (BAF) restored the reduced HER2 levels (Fig. 3C), suggesting that Melatonin may induce HER2 downregulation primarily through the endocytic lysosomal degradation.

Receptors can be internalized from plasma membrane and subjected to degradation by the endosomal-lysosomal pathway [26, 28–30]. To examine the potential role of Melatonin in HER2 endocytosis, we first examined the effect of Melatonin on the cellular localization of HER2 protein in HER2^+^ breast cancer cells. While HER2 protein was expressed strongly on the membrane and diffused in the cytoplasm of the untreated HCC1954 cells, HER2 appeared as intracellular puncta in the Melatonin-treated cells (Fig. 3D), suggesting that Melatonin may induce HER2 subcellular trafficking. Consistently, flow cytometric analysis revealed that Melatonin treatment markedly reduced the amount of HER2 present on the cell surface in a dose-dependent manner (Fig. 3E and Supplementary Fig. 3). These data implicate a potential role for Melatonin in promoting HER2 endocytosis.

**Fig. 4:**
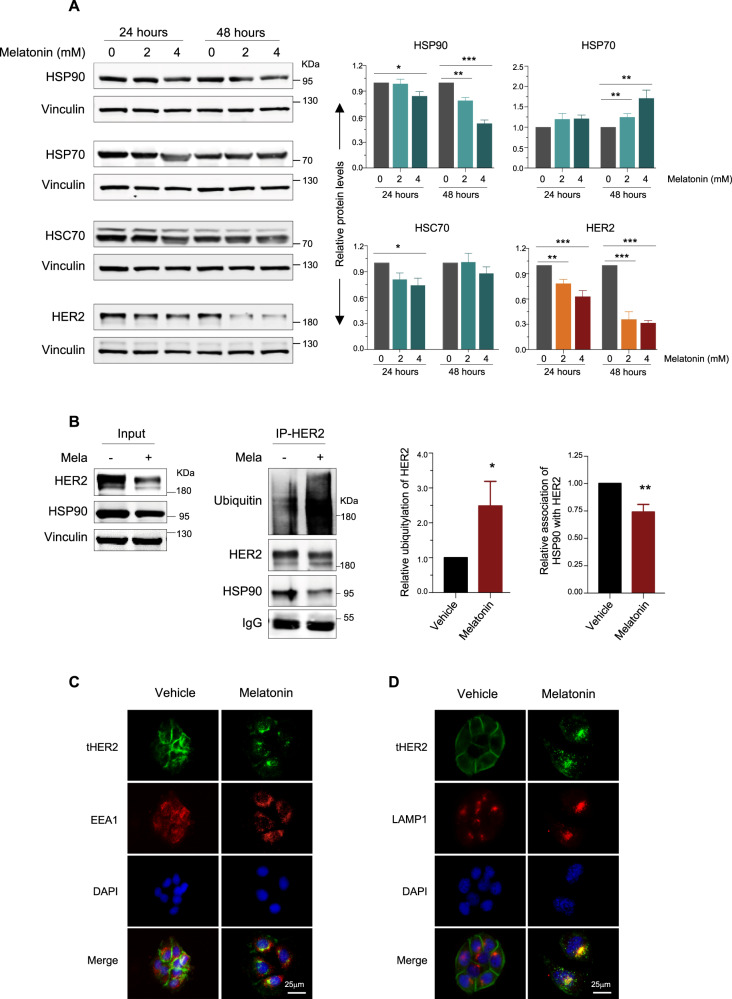
Melatonin induces ubiquitylation of endocytosed HER2 through reducing the expression of HSP90 and its association with HER2. **A** Western blot analysis of proteins as indicated in the HCC1954 cells treated with or without Melatonin. Drug treatment conditions were shown as indicated. Vinculin was used as a loading control. The quantification of protein abundance is shown. **B** Co-Immunoprecipitation (Co-IP) of HER2 and HSP90 from the lysates of the Melatonin-treated HCC1954 cells or control cells. Melatonin, 2 mM, 48 h. Co-immunoprecipitated HSP90 and ubiquitylated HER2 band intensities were quantified and adjusted to the corresponding normalized HER2 signal and shown in the bar charts. Data from three independent experiments are shown as Mean ± S.D. *p < 0.05, **p < 0.01, ***p < 0.001 (Student’s *t* test). C Representative images of immunofluorescent staining of HER2 and EEA1 proteins in the HCC1954 cells treated with 2 mM Melatonin for 24 h. **D** Representative images of immunofluorescent staining of HER2 and LAMP1 proteins in the HCC1954 cells treated as in **C**. Scale bar, 25 μm.

The molecular chaperone system involving HSP90 and HSP70 isoforms (HSP70 and HSC70) has been shown to protect their client protein such as HER2 from ubiquitin-mediated endocytosis [31, 32]. Melatonin treatment resulted in decreased expression of HSP90 and HSC70 and a compensatory increase in HSP70 expression (Fig. 4A), similar to the effect of HSP90 inhibitor 17-AAG [33]. Meanwhile, the amounts of HSP90 co-immunoprecipitated with HER2 were also markedly reduced in the Melatonin-treated cells, suggesting that Melatonin may induce dissociation of HSP90 from HER2 (Fig. 4B). Meanwhile, Melatonin treatment led to increased levels of ubiquitylated HER2 (Fig. 4B). In addition, immunofluorescence staining analysis revealed that Melatonin treatment yielded evident localization of internalized HER2 as puncta structures to endosomal compartment stained with EEA1 (an early endosome marker) (Fig. 4C). Moreover, HER2 was found to colocalize with LAMP1 (a late endosome/lysosomal marker) in the Melatonin-treated HCC1954 cells (Fig. 4D). Together with our finding that the lysosome inhibitor BAF could rescue downregulation of HER2 induced by Melatonin (Fig. 3C), these data support the notion that Melatonin destructs HER2 through promoting its endocytosis and lysosomal degradation.

### Melatonin enhances the cytotoxic effect of Neratinib in HER2^+^ breast cancer cells

**Fig. 5:**
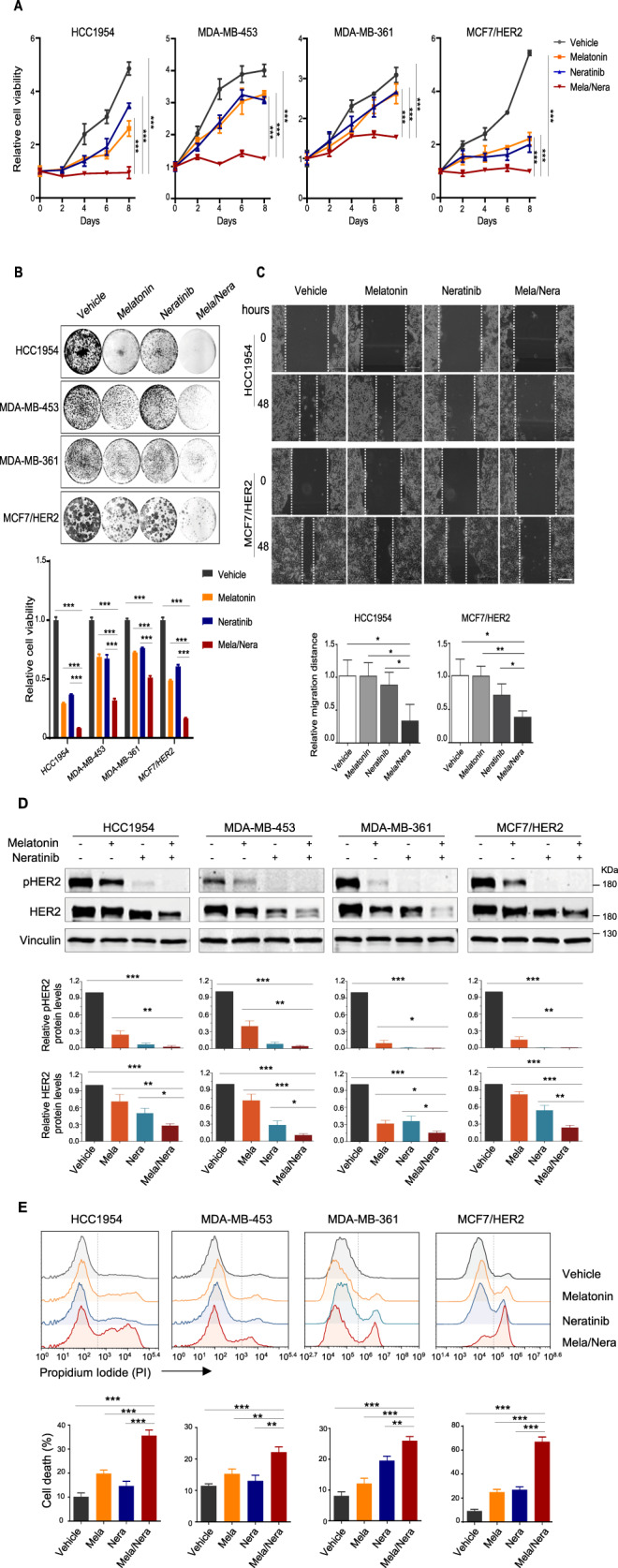
Melatonin synergizes with HER2 inhibitor Neratinib to induce apoptosis and cytotoxicity in vitro. Long-term cell viability of HER2^+^ breast cancer cells treated with Melatonin and Neratinib, either alone or in combination, was examined by crystal violet assay. HCC1954, 2 mM Melatonin, 50 nM Neratinib; MDA-MB-453, 2 mM Melatonin, 100 nM Neratinib; MDA-MB-361, 2 mM Melatonin, 100 nM Neratinib; MCF7/HER2, 1 mM Melatonin, 50 nM Neratinib. **A**, 96-well format; **B**, 24-well format. Representative images of plates and the quantification of cell viability are shown. **C** Cell migration potential of the HER2^+^ breast cancers cells treated as in **A** was determined using a wound-healing assay. The images of the wound areas are shown at 0 and 48 h. Scale bar, 200 μm**. D** Western blot analysis of proteins as indicated in cells treated as in **A** for 24 h. Vinculin was used as a loading control. The quantification of protein abundance is shown. **E** Cell death in the HER2^+^ breast cancer cells treated as in **A** for 48 h was examined by PI staining and FACS analysis. Data from three independent experiments are shown as Mean ± S.D. *p < 0.05, **p < 0.01, ***p < 0.001 (Student’s *t* test).

Built upon our finding that Melatonin decreases HER2 protein stability in this study, we next investigated whether Melatonin could potentiate the therapeutic effect of the pan-HER kinase inhibitor Neratinib in the treatment of HER2^+^ breast cancer cells. To test this hypothesis, we assessed the response of HER2^+^ breast cancer cell lines to these drugs as single-agents or in combination. In all four breast cancer cell lines tested, we observed synergistic treatment response as evaluated by the CalcuSyn model (Supplementary Fig. 4A–C). We further examined the synergistic growth inhibitory effects of the drug combination by clonogenic survival assays. Combined use of Melatonin and Neratinib induced a significantly stronger growth inhibitory effect than either single-agent treatment (Fig. 5A, B). To examine the effect of drug combination on the migration potential of HER2^+^ breast cancer cells, we conducted the wound-healing assays. We found that the gaps between the scratched area were larger in the Melatonin + Neratinib group than those in the single-agent group 48 h post-wounding (Fig. 5C). While single-agent Neratinib [27], and Melatonin to a lesser extent, attenuated HER2 protein abundance as well as phospho-HER2 signal, the combination treatment yielded a more potent inhibitory effect (Fig. 5D). Consistently, while Melatonin or Neratinib alone yielded a moderate cytotoxic effect, the combination treatment resulted in substantially increased cell death (Fig. 5E). Together, these data indicate that the combination of Melatonin and Neratinib exerts synergistic therapeutic activity against HER2^+^ breast cancer cells.

### Combined use of Melatonin and Neratinib effectively blocks the growth of HCC1954 tumor xenografts

**Fig. 6:**
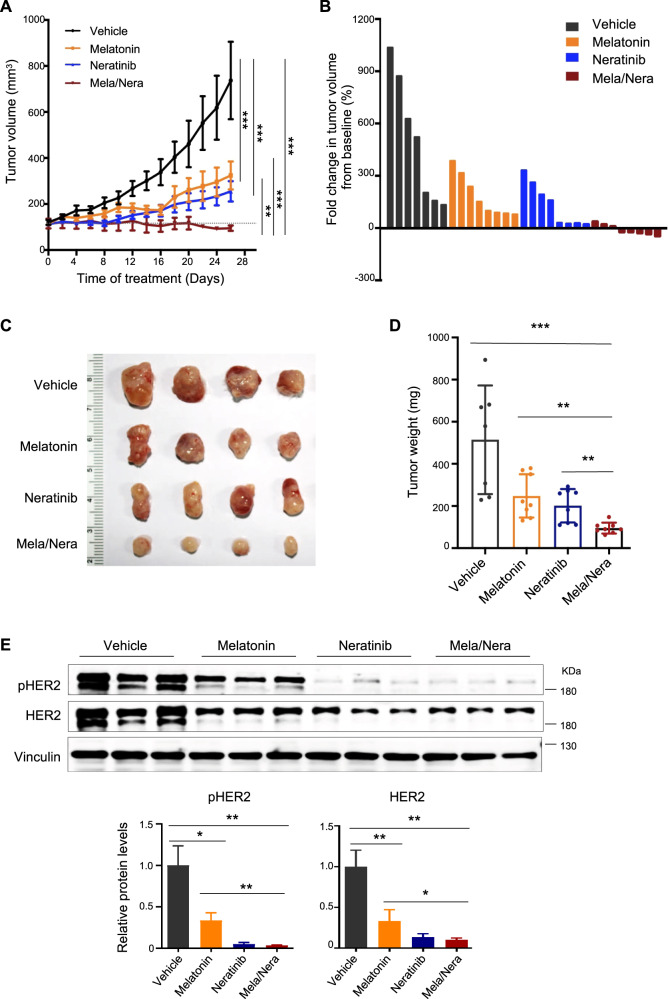
Combined use of Melatonin and Neratinib is effective in vivo. **A** HCC1954 xenograft tumor-bearing mice were treated with Melatonin (50 mg/kg/day, intraperitoneal administration) and Neratinib (5 mg/kg/day, oral gavage), either alone or in combination. Vehicle, *n* = 7; Melatonin, *n* = 8; Neratinib, *n* = 8; Melatonin + Neratinib, *n* = 8. Tumor volume (Mean ± S.E.M) measured at the indicated time points was shown. **p < 0 .01, ***p < 0.001 (Two-way ANOVA with Tukey’s multiple comparison tests). **B** The waterfall plot indicates fold changes in tumor volume of the mice treated as indicated. Fold changes were calculated by (endpoint tumor volume-baseline tumor volume)/baseline tumor volume, multiplied by 100%. Baseline, tumor volume at treatment Day 0. Representative images of tumors (**C**) and tumor weight (**D**) at the endpoint (Day 26) were shown. The data are shown as the mean tumor weight ± S.E.M. **E** The HCC1954 xenograft tumor-bearing mice were treated as in **A** for 3 days and were sacrificed 4 h after the last treatment. Western blot analysis of total- and phospho-HER2 in tumor lysates from the mice treated as indicated. Vinculin was used as a loading control. The quantification of protein abundance is shown. The data are shown as Mean ± S.E.M. (*n* = 3). *p < 0.01; **p < 0.01; ***p < 0.001 (Student’s *t* test).

To validate our in vitro findings, we next evaluated the combinatorial effect of Melatonin and Neratinib in the HCC1954 xenograft tumor model. Compared to the control group, Melatonin and Neratinib monotherapies significantly reduced the tumor growth of the HCC1954 xenografts (Fig. 6A). Strikingly, combined use of Melatonin and Neratinib led to greater inhibition of tumor growth (in terms of average tumor volumes and endpoint tumor weights) than with either agent alone (Fig. 6A–D). Notably, only the combination treatment achieved tumor regression in a fraction of tumors (5 out of 8) (Fig. 6A, B). In keeping with our findings that Melatonin destructed HER2 protein in vitro, the tumors from mice treated with Melatonin alone revealed substantially reduced abundance of HER2 protein (Fig. 6E). Of note, consistent with the recent report [27], we also found that the pan-HER TKI Neratinib decreased HER2 protein abundance as well as HER2 signaling (Fig. 6E and Fig. 5D). In addition, combination treatment did not yield overt toxic effects and mouse body weights were not significantly affected throughout the course of the treatment (Supplementary Fig. 5). Together, these results suggest that combined use of Melatonin and Neratinib may have the potential to improve the treatment response against HER2^+^ breast cancer.

## Discussion

The oncogenic driver HER2 remains a major therapeutic target in breast cancer harboring HER2 amplification/overexpression. Despite the success of HER2-targeted therapy, disease relapse will eventually occur. Intense clinical efforts have been focused on combining HER2-targeted agents of different mechanisms of action or extending the duration of HER2-targeted inhibition. Our study uncovers an unexpected action of Melatonin on destruction of HER2 through inducing HER2 endocytosis and lysosomal degradation. Importantly, our work reveals that Melatonin potentiates the cytotoxic effects of the pan-HER kinase inhibitor Neratinib in HER2^+^ breast cancer both in vitro and in vivo.

The prevention and treatment effects of Melatonin on cancer have been widely studied [14–20]. Previous reports have implicated anticancer properties of Melatonin in HER2^+^ breast cancer [34–38]. Melatonin inhibited cell invasion through suppressing the MAPK/ERK pathway in the studies using HER2^+^ breast cancer cell line modin vitroels [34, 35]. In our study, we showed that Melatonin monotherapy significantly attenuated the growth of HCC1954 xenografts, an effect consistent with previous reports using the MMTV-Neu mouse model of breast cancer [36]. In addition, when co-administered with estradiol-progesterone therapy or Metformin, Melatonin reduced tumor incidence and metastatic burden in the animal model of HER2^+^ breast cancer [36–38]. However, while these and our findings have reported the potential therapeutic effects of Melatonin in the setting of HER2^+^ breast cancer [34–39], the impact of Melatonin on HER2 expression per se has not been previously reported.

In the current study, Melatonin treatment led to significant downregulation of multiple HER2-mediated signal transduction in HER2^+^ breast cancer cells, recapitulating many effects exerted by HER2-targeted agents. These data prompted us to investigate whether Melatonin may inhibit the expression and/or activity of HER2 protein itself. Our work identified an unexpected role of Melatonin in dysregulating HER2 protein stability and potentiating the cytotoxic effect of Neratinib in HER2 + breast cancer. Mechanistically, Melatonin treatment triggered dissociation of HER2 from the HSP90 chaperone complex, and consequently ubiquitin-mediated endocytic trafficking and lysosomal degradation of HER2. Noteworthily, the lysosome inhibitor restored the HER2 protein abundance reduced by Melatonin treatment, whereas the proteasome inhibitor led to accelerated HER2 downregulation (Fig. 3A–C). It has been shown that HER2 ubiquitination is involved in both proteasomal and endocytic degradation [40]. Given that proteasome inhibition results in accumulation of ubiquitylated HER2 [41–43] and that Melatonin induces efficient HER2 ubiquitylation and endocytic lysosomal degradation (Fig. 4B), we reason that the resulting overall increase in HER2 ubiquitylation may accelerate its turnover through the endo-lysosomal degradation pathway [41, 44].

Melatonin is a naturally produced hormone with an impressive long-term safety profile [45–47]. In addition, to enhance therapeutic effects, Melatonin also showed the ability to reduce the adverse effects or general toxicity arising from chemotherapies or radiation in animal models and clinical studies [48–50]. In our study, combined use of Melatonin with Neratinib led to potent inhibitory anti-tumor activities with no overt mouse body weight changes, supporting the potential of Melatonin as a combination adjuvant in the treatment of HER2^+^ breast cancer. Given the prevalence of HER2 positivity in other solid tumors, such as gastric cancer and bladder cancer [51], our finding may expand the potential utility of Melatonin in HER2-targeted therapies beyond breast cancer.

Activated HER2 protein is exclusively present on the plasma membrane in breast cancer cells with HER2 amplification. The HSP90 and its associated molecular chaperone system protects HER2 from degradation, accounting for at least in part the stabilization of HER2 at the plasma membrane [32, 52, 53]. Our data show that Melatonin treatment resulted in decreased levels of HSP90 and HSC70 but a compensatory induction in HSP70 levels. The impairment in the chaperone system arising from Melatonin treatment is indeed consistent with previous reports that HSP70 expression is often concurrently induced in cells following treatment with HSP90 inhibitors or silencing of HSC70 [33, 54]. More importantly, we found that Melatonin treatment also led to attenuated association of HER2 from HSP90. Since HSP90 also chaperones a number of other therapeutic targets, such as CDK4, AKT, and steroid hormone receptors [55–58], it is conceivable that Melatonin may also confer the ability to selectively destruct other oncoproteins chaperoned by HSP90. As such, our study may be broadened to other cancer types with specific actionable vulnerabilities and shed new light on the utility of Melatonin as a promising agent in molecularly targeted cancer therapies.

Neratinib is an irreversible pan-HER receptor TKI. In addition to blocking the activities of pan-HER kinases, Neratinib has also been reported as a HER2 degradation-inducing factor through HER2 endocytosis [27]. In our study, the potential of understanding the activity of Melatonin on HER2 destruction is exemplified by the combinatorial therapeutic effect of Melatonin and Neratinib in HER2^+^ breast cancer cells. Our results from in vitro and in vivo experiments showed that Melatonin significantly enhances the cytotoxicity of Neratinib in a panel of HER2^+^ breast cancer cells harboring PIK3CA mutations known to be less responsive to HER2-targeted agents. Future studies will be needed to examine whether such drug combinations would also be effective in breast cancers resistant to HER2-targeted agents. Collectively, our finding offers a novel dual HER2 blockade (Melatonin plus Neratinib) strategy with effective duration on HER2 destruction.

## Materials and methods

### Cell culture and reagents

All cell lines were obtained from the American Type Culture Collection (ATCC, VA, USA) and maintained in culture medium (HCC1954 and MCF7/HER2 cells in RPMI-1640, MDA-MB-361 and MDA-MB-453 cells in Dulbecco’s Modified Eagle Medium) supplemented with 10% fetal bovine serum (FBS, Biological Industries, Kibbutz, Israel) and 1% penicillin/streptomycin (Gibco, CA, USA) in a humidified incubator at 5% CO_2_ and 37 °C. The cell identity was confirmed by short tandem repeat profiling at the beginning of this study. All cells were negative for mycoplasma during this investigation. Cycloheximide, MG132, Bafilomycin A1, Velcade, Melatonin, and Neratinib were purchased from Med Chem Express (MCE, Shanghai, China).

### Clonogenic survival assay

Cells were seeded on 96-well and 24-well plates, and cultured with or without drugs for days as indicated. Fresh media containing drugs were replaced every other day. At the endpoint, cells were fixed and stained with 0.5% crystal violet solution. Images of stained plates were captured using Epson Scan (Nagano, Japan). Bound crystal violet was resolved by 50% acetic acid solution. The optical absorbance (OD) of bound crystal violet was measured at 570 nm using the Multi-functional microplate reader xMARK^TM^ (Bio-Rad, CA, USA).

### Determination of drug synergy

Cells were plated on 96-well plates and treated with or without drugs for three days, cell viability was assayed using the MTT solution (Aladdin, Shanghai, China). Synergistic effects were determined by the Chou-Talalay method to calculate the combination index [59].

### Western blot analysis

Cell lysates were prepared using ice-cold lysis buffer (50 mM Tris-HCl pH 7.5, 150 mM NaCl, 1% NP-40, 1% sodium deoxycholate, 1 mM C_3_H_7_Na_3_O_6_P, 1 mM NaF, 1 mM Na_4_O_7_P_2_, 1 mM Na_3_VO_4_, and 5 mM PMSF) supplemented with protease/phosphatase inhibitors (Roche, Basel, Switzerland). Western blot experiments were conducted as described previously [60]. The blots were probed with the following primary antibodies: Anti-HER2 (#2165), anti-HSP90 (#4877), anti-phospho-HER2 (Tyr1221/1222, #2243), anti-phospho-AKT (S473, #4060), anti-phospho-ERK1/2 (Thr202/Tyr204, #4370), anti-phospho-NF-κB (Ser536, #3033), and anti-phospho-SRC family (Tyr416, #2101) were obtained from Cell Signaling Technology (CST, MA, USA). Anti-HER2(sc-08) and anti-N-cadherin (sc-271386) were obtained from Santa Cruz Biotechnology (TX, USA). Anti-ubiquitin (10201-2-AP), anti-HSP70 (10995-1-AP), and anti-HSC70 (10654-1-AP) were obtained from Proteintech (Wuhan, China). Anti-E-Cadherin (610181) was obtained from Becton, Dickinson and Company (BD Biosciences, NJ, USA). Anti-Vinculin (V9131) was obtained from Sigma Aldrich (MO, USA). Fluorescent-labeled secondary antibodies against mouse IgG and rabbit IgG were used (Li-COR, Nebraska, USA). Western blots were imaged and quantified by Odyssey Infrared Imaging System (Li-COR, Nebraska, USA).

### Immunoprecipitation

One milligram of whole-cell lysates isolated from the HCC1954 cells treated with or without Melatonin was incubated with anti-HER2 antibody for 4 h at 4 °C followed by the addition of Protein A/G PLUS-Agarose (Santa Cruz Biotechnology) at 4 °C overnight. Beads were washed three times with ice-cold PBS supplemented with protease/phosphatase inhibitors (Roche, Switzerland). The immune complexes were eluted with 2×SDS PAGE sample buffer and analyzed by Western blot.

### Flow cytometric analysis

Cell death assays were performed by using the Propidium Iodide (PI) solution (Dojindo Molecular Technologies, Kumamoto, Japan) according to the manufacturer’s protocol. Briefly, following drug treatment, cells were harvested and stained with PI solution in dark for 15 min. For flow cytometric analysis of HER2 protein levels present on the cell surface, cells were stained with APC anti-HER2 antibody (324407, Biolegend, CA, USA) or APC mouse IgG1 Isotype control antibody (400119, Biolegend). Stained cells were analyzed on NovoCyte Fluidics Station II (Agilent, CA, USA).

### Wound-healing assay

Cells were seeded in 12-well plates and grown until a confluent state. The cells were scratched to generate an artificial wound by using sterile pipette tips. The cell monolayer was then rinsed with PBS three times to remove cell debris. Fresh culture medium with or without drug was added. Images of wound were captured by phase-contrast microscope (Leica, Wetzlar, Germany). The mean width of each scratch was measured using ImageJ software.

### Immunofluorescence staining analysis

Immunofluorescence staining was performed as previously described [61]. Briefly, cells were fixed with 4% formaldehyde, permeabilized with 0.1% Triton X-100, and then blocked in 5% BSA. Then cells were incubated with anti-HER2 (ab134182, Abcam), anti-LAMP1 (#15665, CST) or anti-EEA1 (sc-137130, Santa Cruz) antibodies at 4 °C overnight followed by staining with fluorescence-conjugated secondary antibodies and DAPI solution (Sigma-Aldrich, Missouri, USA). Cells were photographed with a fluorescence microscope (Leica, Germany).

### Quantitative reverse transcription PCR (qRT-PCR)

Total RNA was isolated using NucleoZOL Reagent (Macherey-Nagel, Düren Germany) according to the manufacturer’s instructions. For gene expression analysis, reverse transcription reaction was performed using total RNA by the cDNA synthesis kit (TaKaRa, Dalian, China), and gene expression levels were analyzed by qRT-PCR using SYBR Select Master Mix (Monad, Shanghai, China) in the QuantStudio™ 5 Real-Time PCR system (Thermo Fisher, MA, USA). The relative expression levels of target genes were normalized to *ACTB* and assessed by the delta-delta-Ct (ΔΔCT) method (expressed as 2^−ΔΔCT^). The following primers were used:

*ACTB*

5′-CATGTACGTTGCTATCCAGGC-3′ (Forward)

5’-CTCCTTAATGTCACGCACGAT-3′ (Reverse)

*HER2*

5′-CCGAGGGCCGGTATACATTC-3′ (Forward)

5′-TGCTGTCACCTCTTGGTTGT-3′ (Reverse)

### Analysis of RNA sequencing data

We recently deposited the RNA-seq dataset to the Gene Expression Omnibus (GEO) with accession number GSE175906 [39]. Heatmap showing the expression of leading-edge subsets of gene sets using Omicshare (https://www.omicshare.com/tools/Home/Soft/heatmap). Parametric *t* test *p* values and false discovery rate (FDR) values were reported for each gene. GSEA was performed by the JAVA program (http://software.broadinstitute.org/gsea/index.jsp) using Molecular Signatures Database. 1000 random sample permutations were carried out, and the significance threshold was set at *p* < 0.05 and nominal FDR < 0.05.

### In vivo mouse xenograft study

All the animal experiments were carried out in accordance with the approval of the Animal Research Committee of Dalian Medical University. Eight-week-old female NOD/SCID mice (Vital River Laboratory Animal Technology Co. Ltd, Beijing, China) were maintained in a pathogen-free environment. 5 × 10^6^ HCC1954 cells mixed with Matrigel (BD Biosciences, NJ, USA) were inoculated into NOD/SCID mouse mammary fat pad. Tumor-bearing mice were randomized to four treatment groups (Vehicle, Melatonin, Neratinib, and Melatonin + Neratinib) so that the average starting tumor volumes (about 120 mm^3^) among different groups were similar before drug treatment. Neratinib was dissolved in 0.5% methylcellulose with 0.4% Tween-80 and administered via oral gavage at 5 mg/kg/day. Melatonin was dissolved in ethanol (13%) and administered via intraperitoneal administration at 50 mg/kg/day. Mouse body weights were measured daily during the course of treatment. Tumor volumes were measured every other day with a digital caliper and calculated according to the following formula: tumor volume = (length × width^2^)/2.

### Statistical analysis

The in vitro data are expressed as Mean ± S.D. from three independent experiments. The in vivo data are expressed as Mean ± S.E.M. The unpaired student’s *t* tests and the two-way ANOVA with Tukey’s multiple-comparisons tests were performed using GraphPad Prism software for analysis of the data as specified in the corresponding figure legends. *p* < 0.05 was considered as statistical significance.

## Supplementary information

Supplementary Figures
