# Supplementary material for: Melatonin potentiates the cytotoxic effect of Neratinib in HER2+ breast cancer through promoting endocytosis and lysosomal degradation of HER2
Source: Oncogene. 2021 Sep 23;40(44):6273–83. doi: 10.1038/s41388-021-02015-w (PMC8566236; doi:10.1038/s41388-021-02015-w)
Supplement: Supplementary file 1 — Supplementary Figures [file 41388_2021_2015_MOESM1_ESM.pdf]

Supplementary Figure 1

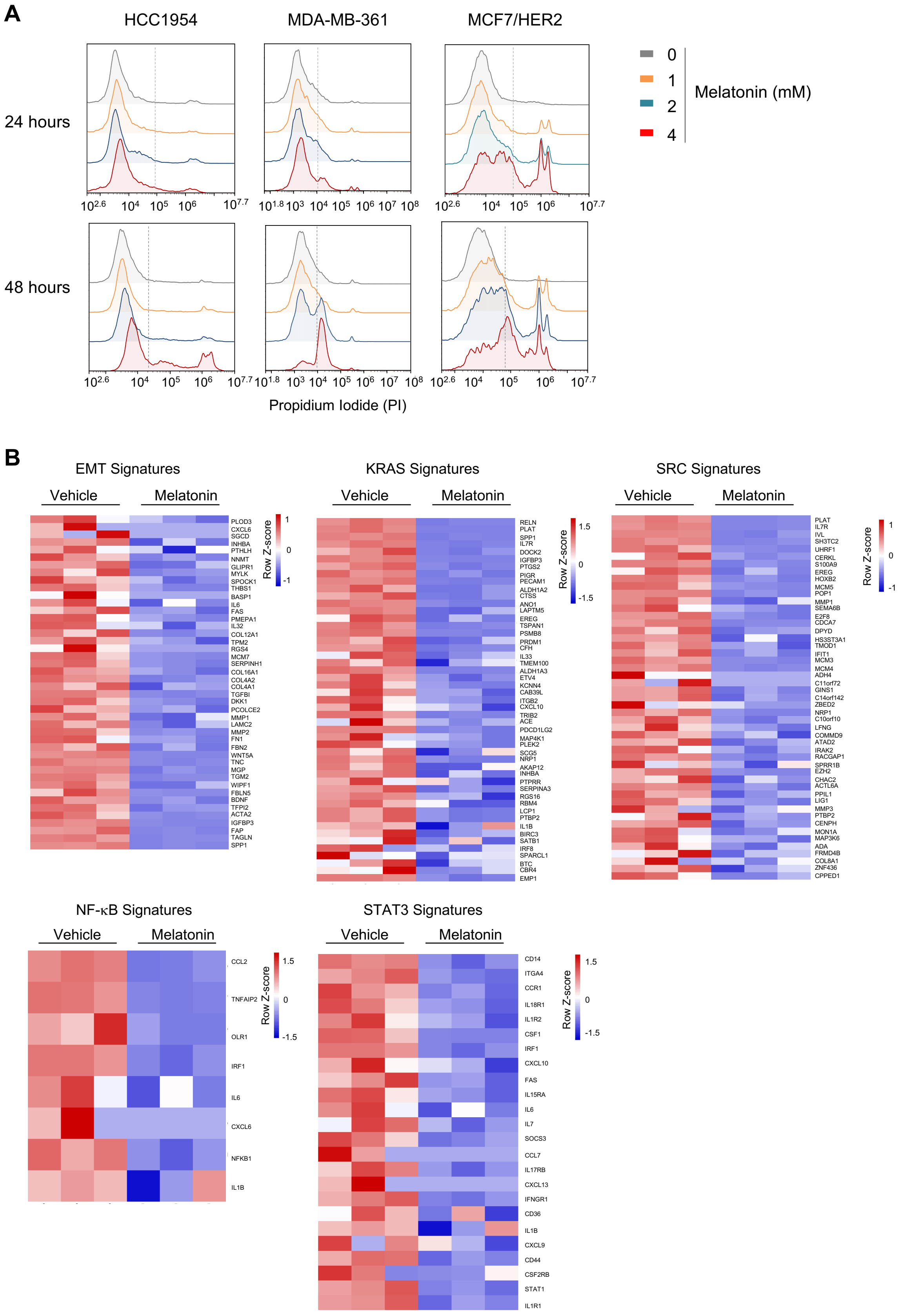

Supplementary Fig. 1. Melatonin treatment increased cell death in HER2+ breast cancer cells. A Cell death in HER2+ breast cancer cells treated with or without Melatonin was determined by PI staining and FACS analysis. Drug treatment conditions were shown. **B** Heatmap showing the expression of leading-edge subsets of gene sets (EMT, KRAS, SRC, NF-κB and IL6-JAK-STAT3) in HCC1954 cells treated with Melatonin or Vehicle control cells. Melatonin, 2 mM, 24 hours.

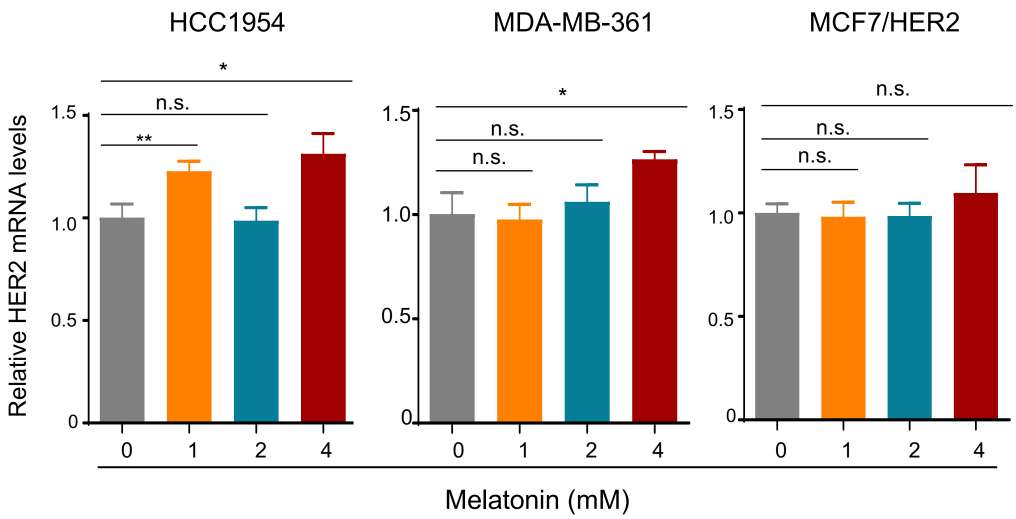

Supplementary Fig. 2. Effects of Melatonin treatment on HER2 mRNA levels in HER2$^+$ breast cancer cells. Quantitative reverse transcription PCR (qRT-PCR) analysis of HER2 mRNA expression in HER2$^+$ breast cancer cells treated with or without Melatonin at the indicated concentrations for 24 hours. *ACTB* was used as an endogenous control. The data are shown as the Mean ± S.D. for three independent experiments. n.s. not significant; * $p < 0.05$, ** $p < 0.01$, (Student's *t*-test).

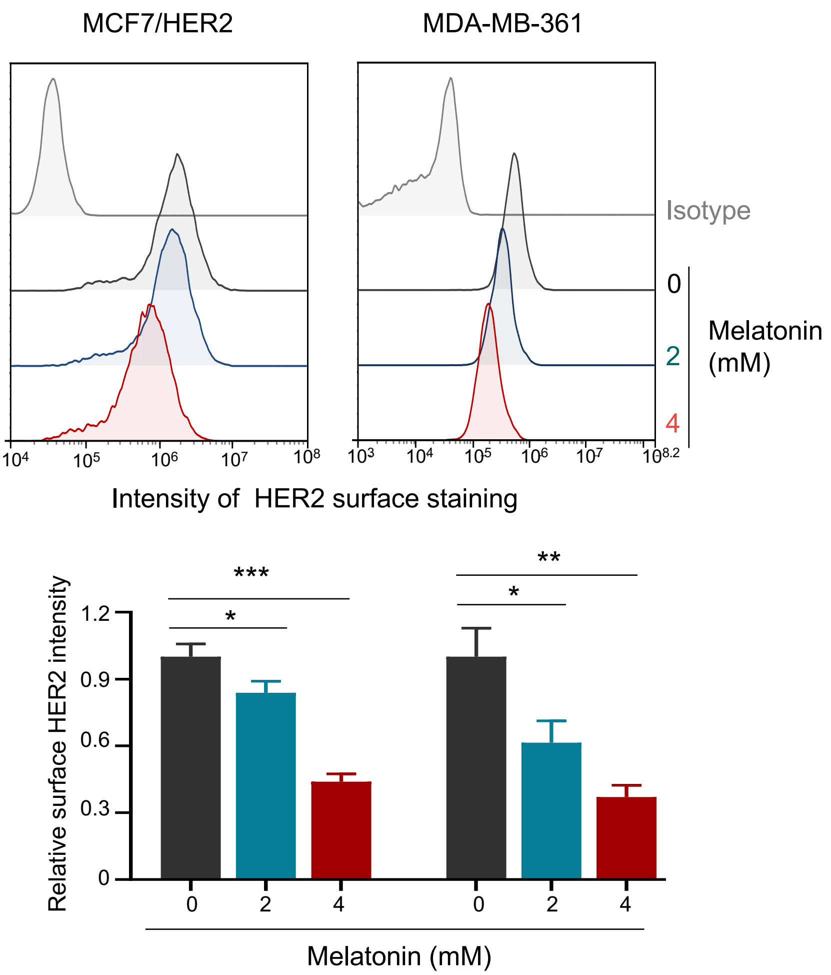

Supplementary Fig. 3. Melatonin treatment reduced the amount of HER2 protein present on the cell surface. Flow cytometric analysis of HER2 protein levels on the surface of cells treated with Melatonin at concentration as indicated for 24 hours. Quantification of HER2 abundance from three independent experiments is shown as Mean ± S.D. * $p < 0.05$, ** $p < 0.01$, *** $p < 0.001$ (Student's *t*-test).

Supplementary Figure 4

A

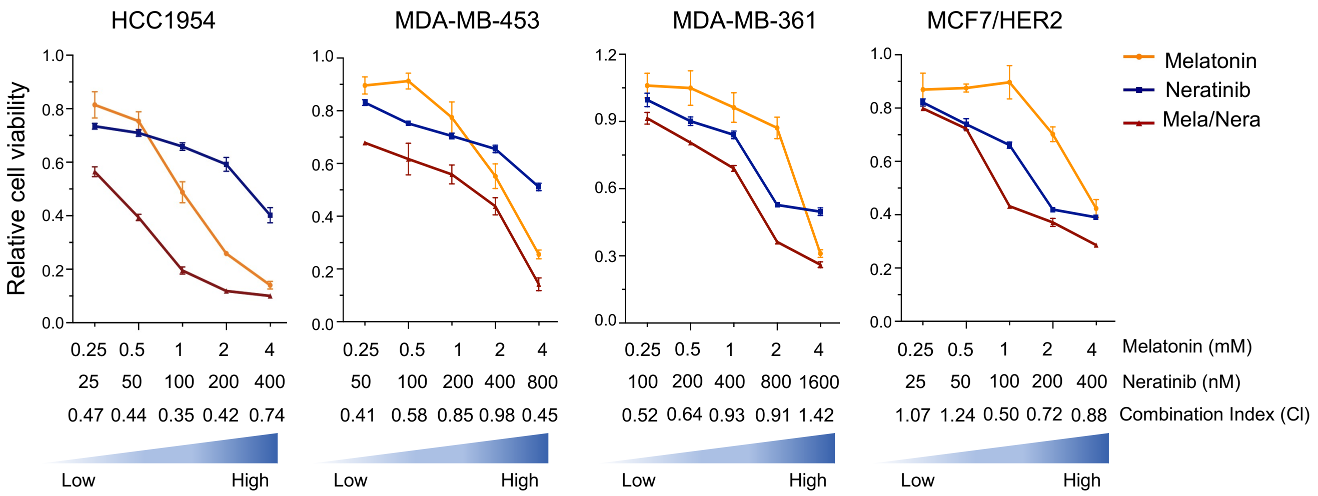

B

		p values							
Cell lines		HCC1954		MDA-MB-453		MDA-MB-361		MCF7/HER2	
		Mela vs. Combo	Nera vs. Combo	Mela vs. Combo	Nera vs. Combo	Mela vs. Combo	Nera vs. Combo	Mela vs. Combo	Nera vs. Combo
Low	Conc. 1	0.00176	0.00139	0.00041	0.00026	0.02496	0.10850	0.22832	0.29043
	Conc. 2	0.00010	0.00005	0.00937	0.09122	0.00599	0.01193	0.00150	0.50142
	Conc. 3	0.00038	0.00002	0.01242	0.01811	0.00258	0.00241	0.00023	0.00009
	Conc. 4	0.00011	0.00006	0.05488	0.00363	0.00006	0.00001	0.00012	0.05269
High	Conc. 5	0.00931	0.00044	0.01195	0.00020	0.03885	0.00155	0.00285	0.00079

C

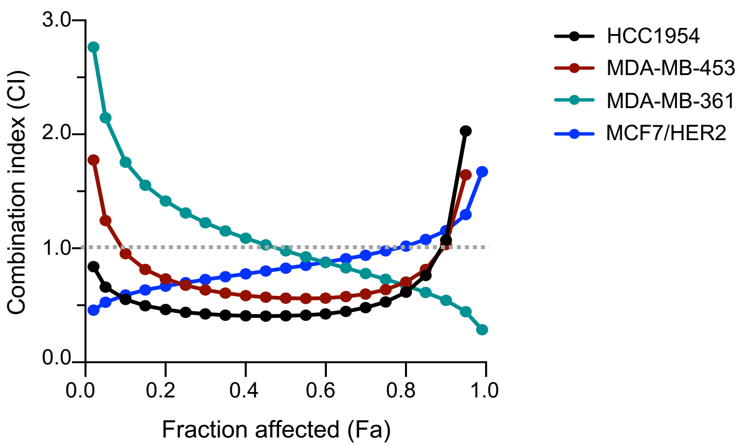

Supplementary Fig. 4. Effects of Melatonin and Neratinib as single-agents or in combination on the growth of $HER2^+$ breast cancer cells. A Dose-response curves of $HER2^+$ breast cancer cells treated with Melatonin and Neratinib, either alone or in combination, at varying concentrations as indicated for 72 hours. The data are shown as Mean ± S.D. Combination index (CI) was calculated using CalcuSyn software with the Chou-Talalay equation. **B** The quantification of cell viability was shown**.** p < 0.05 was considered as statistical significance (Student's *t*-test). **C** The synergistic effect of (**A**) was analyzed using the CI equation and presented with Fa combinations.

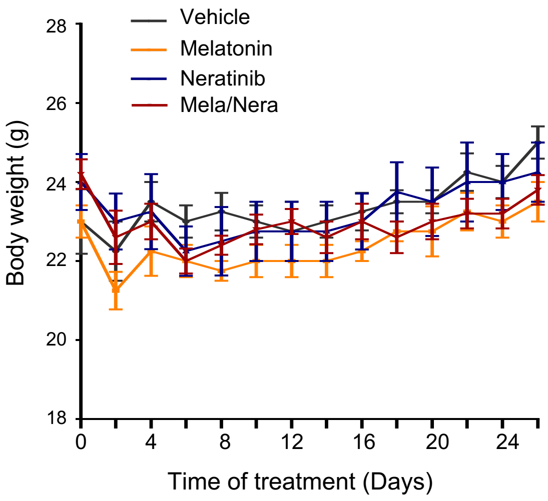

Supplementary Fig. 5. Combined use of Melatonin and Neratinib did not yield overt toxic effects. HCC1954 xenografted mice were treated with Melatonin (50 mg/kg/day, intraperitoneal administration) and Neratinib (5 mg/kg/day, oral gavage), either alone or in combination. Mouse body weight curves of tumor-bearing mice were shown for each treatment group. The data are shown as the Mean ± S.E.M.
